# Cord Blood Cytokine Levels Correlate With Types of Placental Pathology in Extremely Preterm Infants

**DOI:** 10.3389/fped.2021.607684

**Published:** 2021-03-11

**Authors:** Hussein Zein, Khorshid Mohammad, Lara M. Leijser, Marie-Anne Brundler, Adam Kirton, Michael J. Esser

**Affiliations:** ^1^Section of Neonatology, Department of Pediatrics, University of Calgary, Calgary, AB, Canada; ^2^Departments of Pathology and Laboratory Medicine and Pediatrics, University of Calgary, Calgary, AB, Canada; ^3^Section of Pediatric Neurology, Department of Pediatrics, University of Calgary, Calgary, AB, Canada

**Keywords:** cytokines, inflammation, placental pathology, chorioamnionitis, pre-term infants

## Abstract

**Background:** Placental abnormalities are associated with inflammation and have been linked to brain injury in preterm infants. We studied the relationship between placental pathology and the temporal profiles of cytokine levels in extremely pre-term infants.

**Study Design:** We prospectively enrolled 55 extremely preterm infants born between June 2017 and July 2018. Levels of 27 cytokines were measured in blood drawn from the umbilical artery at birth and from infants at 1–3 and 21–28 days of life. Placental pathology was grouped as normal (N), inflammation (I), vasculopathy (V), or combined vasculopathy and inflammation (V+I).

**Results:** Complete data was available from 42 patients. Cord blood median levels of cytokines differed between groups with the highest levels observed in group V+I as compared to groups N, I and V for the following: Eotaxin (*p* = 0.038), G-CSF (*p* = 0.023), IFN-γ (*p* = 0.002), IL-1ra (*p* < 0.001), IL-4 (*p* = 0.005), IL-8 (*p* = 0.010), MCP-1 (*p* = 0.011), and TNFα (*p* = 0.002). *Post-hoc* analysis revealed sex differences between and within the placental pathology groups.

**Conclusion:** Specific types of placental pathology may be associated with differential cytokine profiles in extremely pre-term infants. Sampling from cord blood may help assess the pathological status of the placenta and potentially infer outcome risks for the infant.

## Introduction

Despite advances in neonatal care, pre-maturity continues to be a major cause of mortality and morbidity in children under 5 years of age (1). Although the survival rate of extremely pre-term infants born <29 weeks gestational age (GA) has improved dramatically, rates of morbidity and impaired neurodevelopment continue to be high (2–4). This is problematic as the rates of viable pre-term birth are increasing (5), which has an impact on the child as well as imposing significant familial and socioeconomic stressors (6). Besides, extremely pre-term infants are born at a stage where colonization of the immune cells is ongoing (7), and they are exposed to environmental conditions different from the protective normal *in-utero* environment during the second and third trimester; a key neurodevelopmental period that is highly susceptible to environmental influences.

The placenta is a dynamic feto-maternal organ, and the role of the maternal-fetal unit (mother-placenta-fetus) is complex and interactive, so the effect of one compartment on outcomes may not be straight forward or linear. Clinical studies have repeatedly demonstrated an association between placental abnormalities and neurodevelopmental outcomes in term and pre-term infants (8–11).

Intrauterine inflammation resulting from abnormal placental pathology, like chorioamnionitis, is associated with fetal inflammatory response characterized by high inflammatory cytokines. Cytokines, chemokines, and growth factors (hereafter referred to as cytokines) play important roles in inflammation and cell signaling. They also can influence the various stages of brain development (12–15). Animal studies have shown that systemic inflammation may negatively impact the developing brain (16), and epidemiological studies linking maternal infection to neurodevelopmental abnormalities suggest a risk association with abnormal long-term outcomes such as autism and cerebral palsy (17).

The presence of placental pathology other than chorioamnionitis with or without funisitis can trigger an inflammatory response in the fetus. There is therefore a pressing need to study the association between various types of placental pathology and cytokine profiles in pre-term infants. This may help improve our understanding of the link between different types of placental pathology and the increased risk of brain injury or neurodevelopmental deficits in the absence of histologic chorioamnionitis.

The main objective of this study was to evaluate the relationship between placental pathology and the temporal profiles of cytokine levels in extremely pre-term infants. We hypothesized that cytokine profiles drawn from extremely pre-term infants would correlate with abnormalities seen on placental pathology and that the patterns of cytokines will reflect the different pathologies. A secondary objective was to describe the trends of cytokines' expression over time in these infants.

## Methods

### Population

Extremely preterm infants born between 23 weeks and 0 days to 28 weeks and 6 days GA, admitted to the Level III neonatal intensive care unit (NICU) at Foothills Medical Center, Calgary, Alberta, between June 2017 and July 2018, were prospectively enrolled. Infants with suspected or confirmed congenital and chromosomal abnormalities, congenital infections, and severe intrauterine growth restriction defined as less than the third percentile on the Fenton growth chart (18) were excluded from the study. Written informed consent was obtained from parents before enrollment. Research ethics board approval from the University of Calgary was obtained before enrollment.

### Clinical Data Collection

Relevant pregnancy, birth, and neonatal data were collected from the medical records including maternal age, gravidity, history of smoking, alcohol or illicit drug use during pregnancies, diabetes (gestational or chronic), hypertension (pregnancy-induced or chronic), assisted conception, multiple pregnancy, antenatal steroids, antepartum hemorrhage (defined as any bleeding from the genital tract after the 20th week of pregnancy and before the onset of labor), preterm premature rupture of membranes (PPROM) >24 h, clinical chorioamnionitis (19) and mode of delivery. Neonatal data included: GA, birth weight, sex, Apgar scores, Score for Neonatal Acute Physiology II with Perinatal Extension (SNAP II PE), place of birth (outborn status), respiratory distress syndrome, surfactant administration, bronchopulmonary dysplasia (BPD) (defined as requiring supplemental oxygen or any respiratory support inclusive of nasal Continuous Positive Airway Pressure, or nasal flow cannula ≥1.5 L/min, with or without oxygen, to maintain acceptable SpO_2_), sepsis (early and late-onset), patent ductus arteriosus (PDA), intraventricular hemorrhage (IVH), cystic periventricular leukomalacia, and retinopathy of prematurity.

Blood was collected from enrolled infants at three timepoints: timepoint 1 (T1) from the umbilical cord at birth, timepoint 2 (T2), from the infants between 24 and 72 h of birth, and timepoint 3 (T3), from the infants, between 21 and 28 days of birth. Arterial umbilical cord blood was collected in silicone-coated serum tubes, without additives, and initially stored at 4°C until consent was obtained. Once the infant was enrolled, its cord blood sample was centrifuged at 1,000 g, and the separated serum was aliquoted into cryovials and stored at −80°C until analysis. At T2 and T3, 0.4 ml of blood was collected in lithium heparin tubes, and samples were centrifuged at 3,700 g; the separated plasma was aliquoted in cryovials and stored at −80°C. All samples were stored in the Alberta Children's Hospital BioCORE facility until assayed. To minimize additional samplings from the infants, blood was collected concurrently with routine and clinically indicated blood sampling. Most of the samples, at T2 and T3, were taken from heel pokes (capillary), with only a few taken by venous route.

### Multiplex Assays

Cytokine levels were assayed using the Bio-Rad Multiplex Immunoassay System, which uses microbead suspension array technology (Bio-Rad, Hercules, CA, USA). Relative cytokine levels were generated using the commercially available and standardized plate Bio-Plex Pro™ Human Cytokine 27-plex Assay (#M500KCAF0Y). The targeted cytokines included: Interleukin (IL)−1 beta (IL-1β), IL-2, IL-4, IL-5, IL-6, IL-7, IL-8, IL-9, IL-10, IL-12p70, IL-13, IL-15, IL-17A, interleukin-1 receptor antagonist (IL-1ra), Eotaxin, basic fibroblast growth factor (FGF-basic), granulocyte-colony stimulating factor (G-CSF), granulocyte-macrophage colony-stimulating factor (GM-CSF), interferon-gamma (INF-γ), interferon-inducible protein-10 (Ip-10), monocyte chemoattractant protein-1 (MCP-1), macrophage inflammatory protein-1 alpha (MIP-1α), macrophage inflammatory protein-1 beta (MIP-1β), platelet-derived growth factor-BB (PDGF-BB), Regulated on Activation, Normal T Cell Expressed and Secreted (RANTES), tumor necrosis factor-alpha (TNF-α), vascular endothelial growth factor (VEGF).

### Neuroimaging

Head ultrasound scans were performed as per standard clinical protocol and normal care of very preterm infants. The scans were assessed by a neuroradiologist who was unaware of the clinical course of the infants. IVH grading was in accordance with that described by Papile (20) and designated based on the highest grade in the first week after birth or the first ultrasound if it was done after 7 days of age. Severe IVH was defined as grades III or IV.

### Placental Pathology

After receiving the placentas fresh from the labor and delivery ward, the pathologists assessed the membranes, measured the cord, and documented its insertion, length, diameter, and appearance. They also weighed and measured the disc, after removing the cord and membranes, and again documented the appearance of the disc with the fetal and maternal surface. For microscopy, the following sections were taken: two cross-sections of the cord, one membrane roll, and sections of the central placental disc, and in areas of any lesions identified. The sections for histology were fixed in 10% buffered formalin and processed overnight for histology and stained with Hematoxylin and Eosin. Placental slides were read and graded as per the Amsterdam consensus paper (21) by an experienced placental pathologist (MAB), who was unaware of the clinical course and outcomes of the enrolled infants.

Inflammatory findings in the placenta included maternal inflammatory response (MIR) and fetal inflammatory response (FIR), and both were further divided into three stages per Khong et al. (21). MIR stages included stage 1: acute subchorionitis or chorionitis, stage 2: acute chorioamnionitis, and stage 3: necrotizing chorioamnionitis. FIR stages included stage 1: chorionic vasculitis or umbilical phlebitis, stage 2: involvement of umbilical vein and one or more umbilical arteries, and stage 3: necrotizing funisitis. Vasculopathy was divided into maternal vascular malperfusion and fetal vascular malperfusion. Maternal vascular malperfusion included infarcts, villous development, decidual vaculopathy, and retroplacental hemorrhage, while fetal vascular malperfusion included thrombosis, intramural fibrin, stem vessel obliteration, vascular ectasia, avascular villi, and stromal vascular karyorrhexis.

Finally, placental findings were grouped into (1) normal (N), (2) evidence of inflammation (I), (3) presence of vasculopathy (V), (4), and combined pathology of vasculopathy and inflammation (V+I).

### Statistical Analysis

Data were analyzed using SPSS version 25 (IBM SPSS Statistics for Windows, Version 25.0. Armonk, NY: IBM Corp.). With the annual admission of 120–130 extremely preterm infants to our NICU, and the inability to do a power calculation for our study, we used a convenient sample and recruited as many participants as possible. Descriptive statistics were used to summarize neonatal and maternal demographic characteristics, and data were presented as medians and interquartile range (IQR). After testing for normality (SPSS), the Kruskal-Wallis H test was conducted to determine if there were differences in cytokine levels between the four groups of placental pathology. Subsequently, pairwise comparisons were performed using Dunn's procedure. A Friedman test was run to determine if there were differences in cytokine levels between the three timepoints of sampling. Bonferroni corrections for multiple comparisons were made, and statistical significance was set at *P*-value < 0.05.

## Results

**Figure 1:**
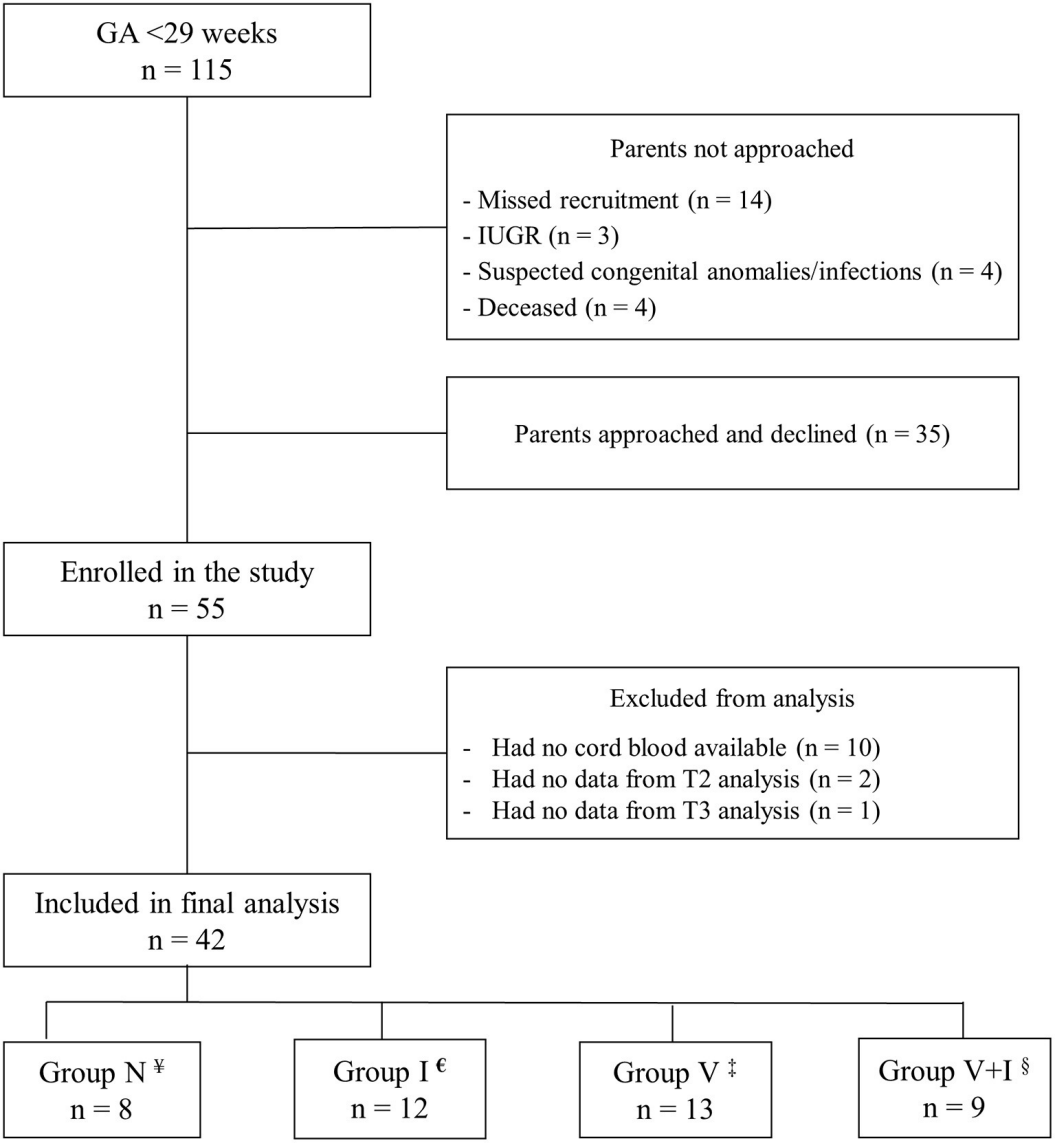
Flowchart of enrollment. ^¥^Normal placenta,^€^Placental inflammation, ^‡^Placental vasculopathy changes, ^§^Combined vasculopathy and Inflammation changes.

Of the 115 extremely preterm infants admitted to our NICU during the study period, 55 (48%) were enrolled in our study (Figure 1). Thirteen infants were excluded from the analysis because of missing at least one timepoint blood sample measurement. There were no differences between the excluded and included patients in terms of GA (*p* = 0.984), birth weight (*p* = 0.255), and SNAP II PE (*p* = 0.169).

The median (IQR) GA was 26 (25–27) weeks, and the median (IQR) birth weight was 840 (682-1,032) grams. There were no significant differences between the demographic (Table 1) and clinical characteristics of the infants included in the analysis except in the case of surfactant administration, where more infants in N and V groups received surfactant compared to those in I and V+I groups (*p* = 0.018). Two infants had severe IVH, one from group V and the other from group V+I. One infant had grade III IVH developed bilateral cystic periventricular leukomalacia and died after the withdrawal of care at 6 weeks of age. Another infant with severe BPD died after discharge from the NICU. None of the infants included in the final analysis had early-onset sepsis or pneumothorax.

**Table 1:** Neonatal, prenatal, and obstetrical characteristics between placental pathology groups.

**Characteristics^†^**	**Placental pathology group**				***P*-value**
	**N^¥^** ** (*n* = 8)**	**I^€^** ** (*n* = 12)**	**V**^‡^ ** (*n* = 13)**	**V+I^§^** ** (*n* = 9)**	
GA, weeks, median (IQR)	26 (25–27)	26 (25–27)	26 (24–27)	27 (24–27)	0.469
Birth weight (g), median (IQR)	926 (721.5–1346.3)	855 (778–1136)	780 (617.5–990)	800 (585–1039.5)	0.368
Male sex	7 (88)	5 (41.7)	8 (62)	6 (67)	0.222
5 min Apgar, median (IQR)	7 (5–8)	8 (7–9)	7 (6–9)	6 (6–8)	0.642
SNAP II PE, median (IQR)	35.5 (20.3–41.8)	27.5 (11–32.8)	19 (10–34)	18 (8–64.5)	0.640
Outborn	1 (12.5)	0	2 (15.4)	1 (11.1)	0.596
Maternal age, median (IQR)	32 (26.5–33.8)	31 (27–33.8)	31 (24.5–36.5)	31 (23.5–36)	0.985
Gravidity, median (IQR)	3 (1.3–4)	2 (1.3–3.8)	1 (1–2)	1 (1–4.5)	0.161
Smoking	2 (25)	0	0	2 (22.2)	0.088
Alcohol	3 (37.5)	0	1 (7.7)	0	0.023
Illicit drugs	1 (12.5)	0	0	2 (22.2)	0.148
Diabetes	0	2 (16.7)	1 (7.7)	1 (11.1)	0.653
Hypertension	0	0	5 (38.5)	1 (11.1)	0.013
Assisted conception	1 (12.5)	2 (16.7)	3 (23.1)	2 (22.2)	0.941
Multiple pregnancy	3 (37.5)	1 (8.3)	4 (30.8)	0	0.115
Antenatal steroids	6 (75)	12 (100)	13 (100)	9 (100)	0.03
Antepartum hemorrhage	6 (75)	3 (25)	1 (7.7)	2 (22.2)	0.009
PPROM > 24 h	3 (37.5)	5 (41.7)	1 (7.7)	5 (55.6)	0.099
Clinical chorioamnionitis	0	3 (25)	0	1 (11.1)	0.134
Cesarean section	6 (75)	4 (33.3)	9 (69.2)	5 (55.6)	0.203

† *Data presented as frequencies with percentages*.

¥ *Normal placenta*;

€ *Placental inflammation*;

‡ *Placental vasculopathy changes*;

§ *Placental vasculopathy + inflammatory changes*.

*SNAP II PE, Score for Neonatal Acute Physiology II Perinatal Extension; PPROM, preterm premature rupture of membranes*.

In terms of maternal variables, hypertension was associated with placentas categorized as having vasculopathy or combined changes (*p* = 0.013), and diabetes was associated with abnormal placentas of all three categories. Antepartum hemorrhage was more common among the group with normal placentas (*p* = 0.009); this group also received less antenatal steroids (75% compared to 100%) than the other three groups (*p* = 0.03).

### Placental Pathology Findings

Histologic findings of the 42 cases were as follows: 8/42 (19%) cases had no significant histologic findings, 12/42 (29%) had acute inflammation, 13/42 (31%) had vasculopathy findings, and 9/42 (21%) had combined inflammation and vasculopathy findings. MIR was seen in 21/42 (50%) of the placental pathology readings, and histologic chorioamnionitis was diagnosed in 15/21 (71%), while chorionitis or acute subchorionitis was seen in the remaining 6/21 (29%) cases. FIR was seen in 18/42 (43%) cases, and all were associated with MIR changes. None of the placentas had findings of chronic inflammation. Maternal vascular malperfusion was more common (19/45, 45%) than fetal vascular malperfusion (6/42, 14%).

### Cytokine Analysis

There were no differences in cytokine levels between cases of acute chorioamnionitis alone or acute chorioamnionitis with FIR at the three timepoints. Also, no significant differences were noted when comparing between the stages of MIR or FIR.

### Cytokine Analysis Between Groups

Median levels of several cytokines differed across the four placental groups (Table 2), and values of significance have been adjusted by Bonferroni correction for multiple tests. At T1, eight cytokines (Eotaxin, G-CSF, IFN-γ, IL-1ra, IL-4, IL-8, MCP-1, and TNF-α) had significantly higher levels between at least two groups. At T2, fewer differences in cytokine (IFN-γ, IL-4, and MCP-1) levels were seen between the four placental groups. There were no significant differences in cytokine levels between the groups at T3.

**Table 2:** Cytokines with significant differences in levels between placental pathology groups.

**Variable^†^**	**Placental pathology groups**				***P*-value**
	**N^¥^**	**I^€^**	**V^‡^**	**V+I^§^**	
**T1 (CORD BLOOD)**					
Eotaxin	14.8 (9.3–18)	16.5 (13.6–33.4)	20.1 (15.4–28.2)	24.2 (21.3–33.6)	0.038
G-CSF	319.1 (239.1–650.5)	613.4 (243.2–1134.7)	241.9 (187.7–924.3)	749.7 (481.2–2311.8)	0.023
IFN-γ	7 (3.6-9)	17.7 (7.4-26.7)	9.5 (6.6-15)	42.4 (17.3-72.8)	0.002
IL-1ra	310.9^*^ (215.6–891.3)	2672.9 (745.3–10350.8)	255.4^*^ (143.9–1092.5)	7312.1^*^ (2312–16131.1)	<0.001
IL-4	0.7 (0.4–0.9)	1.1 (0.7–1.6)	1 (0.8–1.5)	1.6 (1.4–3.5)	0.005
IL-8	37.6 (23–46.1)	112.2 (56.4–240.3)	42 (22.8–131.7)	197.1 (84.1–597)	0.010
MCP-1	79.3 (57.4–148.6)	64.2 (22.1–188.4)	134.4 (80.1–276.7)	238.6 (184.6–476)	0.011
TNF-α	45.3 (30.2–60.9)	84.1 (42.9–156.8)	34.9^*^ (27.7–57.8)	87.7^*^ (63.1–138.9)	0.002
**T2 (24–72 h)**					
IFN-γ	45.3 (23.3–82.3)	24 (12.3–34.8)	79.1 (54–119.1)	68.3 (34.5–86)	0.008
IL-4	1.1 (1–1.5)	0.6 (0.5–1)	1.6 (0.9–2.4)	1.5 (1.1–2.5)	0.017
MCP-1	642.1 (187.5–980.5)	197.4 (87.6–297.7)	963.1 (381.5–1859)	580 (106.8–1210.6)	0.033

*Red font = significantly higher and blue font = significantly lower*.

*T1: Eotaxin median levels were significantly higher in the V+I group compared to N group (p = 0.045). G-CSF median levels were higher in the V+I group compared to the V group (p = 0.019). Median levels of IFN-γ were higher in the V+I group compared to N (p = 0.004) and V (p = 0.011) groups. IL-1ra levels were higher in V+I group compared to V (p = 0.001) and N (p = 0.015) groups, and in the I group compared to the V group (p = 0.041). Levels of IL-4 and IL-8 were both higher in the V+I group compared to the N group (p = 0.002) and (p = 0.049) respectively. MCP-1 levels were higher in the V+I group compared to the I group (p = 0.014) and N group (p = 0.042). TNF-α levels were higher in the I (p = 0.033) and V+I (p = 0.007) groups compared to the V group*.

*T2: The I group had higher levels of IFN- γ (p = 0.005) and MCP-1 (p = 0.021) compared to the V group. When compared to levels in the I group, IL-4 levels were higher in the V (p = 0.038) and V+I (p = 0.033) groups*.

† *Data presented as medians (IQR), pg/ml*.

¥ *Normal placenta*;

€ *Placental inflammation*;

‡ *Placental vasculopathy changes*;

§ *Placental vasculopathy + inflammatory changes. Underlined red values are significantly higher than underlined blue values. Stared red values are significantly higher than stared blue values*.

### Cytokine Analysis Within Each Group Over Time From Birth

Significant differences in cytokine levels over the three sampling timepoints are shown in Supplementary Table 1 in the Supplementary Material.

### Sex Differences in Cytokine Levels

In a *post-hoc* analysis, at T1, males had significant differences in levels of Eotaxin, G-CSF, IFN-γ, IL-1ra, IL-4, IL-8, MCP-1, and TNF-α between the placental pathology groups with the highest levels noted in the V+I group. In females, however, there were no significant differences in cytokine levels (Table 3).

**Table 3:** Differences in cytokine levels as a function of sex and placental pathology at T1.

**Variable^†^**	**Placental pathology-male sex**				***P*-value**
	**N** ***n* = 7**	**I** ***n* = 5**	**V** ***n* = 8**	**V+I** ***n* = 6**	
Eotaxin	13.6 (8.1–17.7)	17.4 (14.2–29.4)	21.9 (14.9–27.5)	25.7 (22.8–57.3)	0.012
G-CSF	327.5 (225.1–691.9)	227.5 (134.5–593.9)	228.8 (204–345.1)	793.9 (483.1–2428.9)	0.02
IFN-γ	6.9 (3.5–8.5)	15.7 (4.7–21.7)	10.1 (8.1–15)	53.6 (25.9–92.9)	0.012
IL-1ra	359.6 (245.7–1061.9)	572.9 (265–1824.5)	236.7 (129.9–768.6)	10373.7 (5866.1–21199.1)	0.005
IL-4	0.6 (0.4–0.9)	0.9 (0.4–1.2)	1 (0.9–1.4)	1.9 (1.5–3.4)	0.003
IL-8	33.2 (22.4–46.7)	56.4 (40.3–81.6)	34.5 (21.6–109.1)	178.9 (103.1–373.5)	0.025
MCP-1	75.6 (53.7–137.6)	56.8 (24–265.1)	199.5 (124.5–290.7)	242.5 (192–998.7)	0.007
TNF-α	48.6 (29.8–62.6)	47.2 (37.4–72.6)	33.4 (27.6–52.9)	89.1 (66.9–142.5)	0.016
**Variable**^†^	**Placental Pathology-female sex**				***P*****-value**
	**N** ***n*** **=** **1**	**I** ***n*** **=** **7**	**V** ***n*** **=** **5**	**V+I** ***n*** **=** **3**	
Eotaxin	22.5	16.1 (13.2–38)	20.1 (11.9–55.5)	23.8 (12.7–25.5)	0.908
G-CSF	310.7	754.7 (606.8–2198.3)	304.8 (146–887.8)	512.1 (450.3–3680.1)	0.312
IFN-γ	9.1	20.9 (9.5–52.7)	8.2 (3.7–17.1)	23.8 (9–78.6)	0.188
IL-1ra	162.5	10098 (2959.1–13019.1)	546.8 (183.6–1687.3)	3075.4 (454.4–16661.1)	0.05
IL-4	0.9	1.2 (0.9–1.8)	1.5 (0.7–2.5)	1.3 (1–3.9)	0.735
IL-8	42	213.1 (117.7–271)	91.4 (47–176.1)	292.6 (26.2–1628.7)	0.209
MCP-1	239.9	145.9 (21.3–200.1)	81.1 (50.2–202.5)	185.4 (176.3–451.9)	0.342
TNF-α	42.1	130.1 (76–192.2)	35.6 (29.3–63.5)	87.7 (43.9–176.1)	0.074

† *Data presented as medians (IQR), pg/ml*.

^¥^*Normal placenta*; ^€^*Placental inflammation*; ^‡^*Placental vasculopathy changes*; ^§^*Placental vasculopathy + inflammatory changes*.

When we examined the data for sex-differences within each placental group (Table 4), we noted the following: in the I group, the levels of FGF-basic, G-CSF, IL1b, IL-1ra, IL-8, MIP-1α, and TNF-α were significantly higher in females compared to males. In the V group, males had higher levels of FGF-basic at T1, IFN-γ at T2, and IL-4, IL-5, and VEGF at T3. In the V+I group, females had higher levels of IL-9 at T1 and males had higher levels of IP-10 at T2.

**Table 4:** Sex differences in specific cytokine levels in the placental groups with pathology.

	**Variable^†^**	**Sex**		***P-*value**
		**Male (*n* = 5)**	**Female (*n* = 7)**	
Group I	**T1**			
	FGF-basic	34.9 (34.4–41.9)	86.5 (40.0–109.2)	0.03
	G-CSF	227.5 (134.5–593.9)	754.7 (606.8–2198.3)	0.048
	IL-1β	0.6 (0.4–1.1)	3.8 (0.9–10.8)	0.038
	IL-1ra	572.9 (265–1824.5)	10098.0 (2959.1–13019.1)	0.005
	IL-8	56.4 (40.3–81.6)	213.1 (117.7–271.0)	0.005
	MIP-1α	6.7 (4.1–9.6)	16.3 (7.5–33)	0.048
	TNF-α	47.2 (37.4–72.6)	130.1 (76–192.2)	0.048
	**T2**			
	TNF-α	71.9 (49.4–81.3)	41.8 (36.9–51.6)	0.018
		**Male (*****n*** **=** **8)**	**Female (*****n*** **=** **5)**	
Group V	**T1**			
	FGF-basic	47.5 (37.4–55)	25.3 (22.2–39.5)	0.019
	**T2**			
	IFN-γ	99.8 (76.2–143.6)	54.7 (37–78.2)	0.045
	**T3**			
	IL-4	2.4 (1.8–3)	1.6 (1.1–1.7)	0.019
	IL-5	25.8 (23.6–34.5)	16.9 (16.2–19.8)	0.017
	VEGF	327.5 (259.2–390.9)	223 (192.7–242.6)	0.019
		**Male (*****n*** **=** **6)**	**Female (*****n*** **=** **3)**	
Group V+I	**T1**			
	IL-9	203.9 (164.8–245.6	273.9 (265.1–331.8)	0.024
	**T2**			
	IP-10	532 (301.9–1100.7)	169 (41–231.3)	0.048

† *Data presented as medians (IQR), pg/ml*.

^¥^*Normal placenta*; ^€^*Placental inflammation*; ^‡^*Placental vasculopathy changes*; ^§^*Placental vasculopathy + inflammatory changes*.

## Discussion

In this study, we found a significant association between different types of placental pathology and blood cytokine (cytokines, chemokines, and growth factors) levels in extremely pre-term infants. Of the 27 cytokines measured, eight showed significant differences between the four groups of placental pathology, and this significance was mostly noted from samples drawn from umbilical cord blood soon after birth. Cytokine levels also varied between the three timepoints within groups and between infants within each placental pathology group over time. Males had significant differences in cytokine levels between placental pathology groups, while females had significantly higher levels of specific cytokines in the I group as compared to males in samples drawn from cord blood. In our study, we also compared cytokine concentrations between different placental pathology groups, where we differentiated between I and V groups and noted higher levels of these cytokines when there was evidence of both vasculopathy and inflammation (V+I) in the placentas. The higher levels of cytokines in the V+I group at T1 suggests an increased effect of placental inflammation when in the context of concomitant vasculopathy; a finding that was more pronounced in males, which makes delineation of other sex differences in biological measures an interesting area of investigation that we are exploring in ongoing research.

The source of measured cytokines at T1 can be fetal, maternal, placental, or a combination. Concerning fetal sources of cytokines, studies examining whether cytokines cross the placenta have shown conflicting results. In 2004, Zaretsky et al. reported on the bidirectional crossing of IL-6 but not of IL-1β or TNF-α (22). However, a second study found that none of these proinflammatory cytokines crossed the placenta (23). It is important to note that both studies were done on term GA placentas taken from healthy mothers after elective cesarean sections and that the placentas had no signs of inflammation or other pathology. Thus, the findings from these studies may not be directly indicative of placental barrier function in cases where there are placental pathology and pre-mature birth as in our study. In pre-clinical studies with rodents, radiolabelled IL-6 has been shown to cross the placenta in mid and late pregnancy, with a striking 15-fold higher unidirectional maternal-fetal clearance for IL-6 in mid-gestation (24). Similar results have been reported for IL-2 using a mouse model (25). In light of these differences, it seems reasonable to infer that the placenta of an extremely preterm infant with pathology is not likely to have the same function as the placenta from a healthy maternal-neonate dyad. As such, the placentas from our cohort could have been the source of elevated cytokine levels in cord blood samples secondary to loss of integrity from inflammation. Higher cytokine levels could also reflect ongoing signaling from the inflamed placenta itself with a subsequent drop in levels after birth secondary to protein degradation in the newborn blood and the loss of inflammatory signal originating from the placenta. In scenarios like this, it is difficult to anticipate how long the fetus would be exposed to these stimuli and how this would affect brain development.

Many of the cytokines in our study can either have a direct pleiotrophic effect on the central nervous system (26, 27) or have been associated with certain behavioral and psychiatric outcomes (28–30). The risk of abnormal neurodevelopmental outcomes was increased in cases of combined histologic chorioamnionitis and perfusion defects in very preterm and extremely low birth weight infants (31). In our study, the levels of several cytokines were significantly higher in the combined pathology of acute inflammation and vascular malperfusion in the placenta. This highlights the importance of pathologies other than acute histologic chorioamnionitis (with or without funisitis) on the cytokines, which are well-known for the significant role they play at different stages of brain development (32–36). More research in this field is required to examine further and explore this finding.

Cytokine stability varies depending on the collection method and storage time before processing, and while cytokine concentrations were reported to be higher in serum compared to plasma (37), serum samples may be more optimum for the detection of analytes (38). Data from Aziz et al. showed a significant increase in concentrations of IL-1ra when left unprocessed and stored at both room temperature and in the refrigerator (4°C) for up to 24 h (39). However, our data were not consistent with these findings and showed higher concentration levels of IL-1ra in the placental pathology groups with inflammatory findings (groups I and V+I). In the I group, the concentration levels of IL-1ra were 8–10-folds higher than N and V groups, and in the V+I group, the concentrations were even higher by 23–28-folds. This significant increase in IL-1ra levels in the groups with inflammatory findings only could represent a direct effect of placental inflammation on IL-1ra expression rather than by the delayed processing times.

One of the interesting findings from our *post-hoc* analysis is the sex-dependent differences in cytokine concentrations that suggest the differences between levels among placental groups at T1 may be partially driven by the male sex. In comparison analysis across placental groups in females showed that the concentrations of G-CSF, IFN-γ, IL-1ra, IL-8, MCP-1, and TNF-α were higher in group I and group (V+I) at T1 as compared to later timepoints, although this did not reach statistical significance, and maybe partially explained by the smaller sample size in each of the pathology groups. This finding may have particular clinical relevance as other studies have shown male sex to be associated with higher risk maternal complications, fetal distress during labor, and increased rates of placental abnormalities (40–42). Further, studies have found an association between male sex and higher rates of mortality and adverse neurodevelopmental outcomes in extremely pre-term infants (43, 44). However, the reasons for these sex differences are not clearly understood, and a potential role of cytokines, chemokines, and growth factors together with hormonal and genetic differences between the two sexes could help to better understand this phenomenon and its potential implications.

### Limitations

While the results of this study are intriguing and are the nidus for future work trying to better understand the pathophysiology underlying modifiers of outcomes, several limitations should be acknowledged. The most significant limitation is that of the small sample size, especially in the context of three different placental pathology groups and the two sexes, which may have contributed to an incomplete appreciation of group or sex differences. Hence, ongoing studies in our program continue recruitment to strengthen our analysis and be able to do further statistical analyses that will help us adjust for confounding factors. Another limitation is the delay in the processing of serum samples drawn from umbilical cord blood. Serum samples were kept unprocessed and refrigerated at 4°C until patients were formally recruited and signed consent in place. Further, the samples were centrifuged at different times relative to being drawn if it were done at 24 or 72 h after birth, which could have affected the stability of cytokines. A third limitation to our study was the use of multiple comparisons, which could have resulted in increased false-positive results. However, we used the Bonferroni correction in these cases.

## Conclusions

Results from this study suggest that placental pathology is associated with differential cytokine concentrations as a function of the type of pathology, infant sex, and time from birth. Further, this type of correlation of cytokine levels to pathology type, in conjunction with what is known about the effect of placental pathology on neurodevelopmental outcomes, could have high utility for prognostication as well as targeted interventions when more definitive information is not available. However, longer outcome analyses will be needed to examine these initial findings in terms of how they relate to the infant's neurodevelopmental trajectory.

## Data Availability Statement

The raw data supporting the conclusions of this article will be made available by the authors, without undue reservation.

## Ethics Statement

The studies involving human participants were reviewed and approved by Conjoint Health Research Ethics Board (CHREB), University of Calgary. Written informed consent to participate in this study was provided by the participants' legal guardian/next of kin.

## Author Contributions

HZ, KM, M-AB, and ME made substantial contributions to conception and design, acquisition of data, or analysis and interpretation of data. M-AB and HZ reviewed the placental pathology slides. HZ and ME drafted the first manuscript. KM, LL, AK, and M-AB contributed to critical revision for important intellectual content and approved the publication's final version. All authors contributed to the article and approved the submitted version.

## Conflict of Interest

The authors declare that the research was conducted in the absence of any commercial or financial relationships that could be construed as a potential conflict of interest.
