# Supplementary material for: Cord Blood Cytokine Levels Correlate With Types of Placental Pathology in Extremely Preterm Infants
Source: Front Pediatr. 2021 Mar 11;9:607684. doi: 10.3389/fped.2021.607684 (PMC7991101; doi:10.3389/fped.2021.607684)
Supplement: Supplementary file 1 [file Table_1.pdf]

frontiers

# *Supplementary Material*

## 1 Supplementary Table 1

**Table 1|** Cytokines with significant differences in levels as a function of sampling timepoint for each of the placental pathology groups.

| Variable† | Collection time point | | | P value |
|---|---|---|---|---|
| | T1 | T2 | T3 | |
| **Group N¥** | | | | |
| **Eotaxin** | 14.8 (9.3-18) | 32.1 (16.5-33.4) | 56.95 (27.7-65.1) | 0.002 |
| **IFN-γ** | 7 (3.6-9) | 45 (23.3-82.3) | 34.3 (22-73.6) | 0.021 |
| **IL-1ra** | 310.9 (215.6-891.3) | 3752.7 (1862-8550) | 3176.9 (907.1-7905.5) | 0.011 |
| **IL-4** | 0.7 (0.4-0.9) | 1.1 (1-1.5) | 1.7 (1.2-2) | 0.011 |
| **MCP-1** | 79.3 (57.4-148.6) | 642.1 (187.5-980.5) | 358.9 (171.4-634.7) | 0.008 |
| **PDGF-BB** | 3649 (1475.3-5351.1) | 269.9 (141.3-442.6) | 1101.1 (462-1390.1) | 0.005 |
| **Group I€** | | | | |
| **Eotaxin** | 16.5 (13.6-33.4) | 16.8 (7.9-25.2) | 52.6 (41.1-65) | 0.002 |
| **G-CSF** | 613.4 (243.2-1134.7) | 148.3 (94.7-580.7) | 317.2 (245.3-453.7) | 0.005 |
| **IFN-γ** | 17.7 (7.4-26.7) | 24 (12.3-34.8) | 42.4 (32.6-49.3) | 0.017 |
| **IL-4** | 1.1 (0.7-1.6) | 0.6 (0.5-1) | 1.8 (1.6-2.1) | 0.006 |
| **IL-6** | 19.6 (8.6-257.6) | 5.6 (4.9-21.6) | 4.3 (3.5-4.8) | 0.007 |
| **IL-9** | 172.56 (142.8-224.3) | 82.8 (52-180.6) | 154.3 (123.8-202.9) | 0.018 |
| **MCP-1** | 64.2 (22.1-188.4) | 197.4 (87.6-297.7) | 417 (332.3-677.4) | 0.001 |
| **MIP-1α** | 9.7 (5.7-19.6) | 3.4 (2-10.2) | 4.9 (2.5-7.4) | 0.005 |
| **MIP-1β** | 132.3 (103-185.9) | 65 (36.6-81.7) | 78.8 (60.2-98.5) | <0.001 |
| **PDGF-BB** | 4286.5 (1617-5572.9) | 244 (93-383.3) | 6110 (4473.8-10160.9) | <0.001 |
| **TNFα** | 84.1 (42.9-156.8) | 37.6 (29-61.8) | 48.8 (41-70) | 0.028 |
| **Group V‡** | | | | |
| **Eotaxin** | 20.1 (15.4-28.2) | 47.2 (27.3-59.7) | 47 (28.6-81.1) | 0.037 |
| **IFN-γ** | 9.5 (6.6-15) | 79.1 (54.1-119.1) | 47.9 (31.9-76.4) | <0.001 |
| **IL-1ra** | 255.4 (143.9-1093) | 4001 (2813-6828) | 2944 (1630-5432) | <0.001 |
| **IL-8** | 42 (22.9-131.7) | 122.9 (55.2-185) | 67.3 (60.2-106.2) | 0.037 |
| **IL-9** | 162.3 (122.1-221.1) | 94.5 (64.3-124.3) | 165.9 (99.9-249.1) | 0.009 |
| **MCP-1** | 134.4 (80.1-276.7) | 963.1 (381.5-1859) | 481.8 (282.9-723.9) | <0.001 |
| **PDGF-BB** | 3091.3 (1639.8-4832.9) | 302 (161-506.4) | 874.5 (398.8-1551.5) | <0.001 |
| **Group V+I§** | | | | |
| **FGF-basic** | 52 (25.8-83.1) | 30.8 (17.1-43.4) | 27.6 (22.6-33.8) | 0.016 |
| **G-CSF** | 749.7 (481.2-2311.8) | 338.2 (127.5-446.7) | 369.7 (271.7-516.7) | 0.018 |
| **IL-9** | 239.3 (186.2-269.5) | 109.8 (49.6-198.8) | 121.2 (79.9-235.6) | <0.001 |
| **MIP-1α** | 8 (6.2-37.5) | 3.9 (3-10.1) | 3.3 (2.5-4.6) | 0.004 |
| **MIP-1β** | 169.4 (107.5-388.1) | 80.7 (53.6-97.3) | 93.5 (45.6-128.9) | 0.008 |

| | | | | |
|---|---|---|---|---|
| **PDGF-BB** | 4491.9 (3533.5-5398) | 342.2 (275.4-568.6) | 658.2 (370.4-1775.6) | 0.001 |

† Data presented as medians (IQR), pg/ml

[¥] Normal placenta; [€] Placental inflammation; [‡] Placental vasculopathy changes; [§] Placental vasculopathy + inflammatory changes

Group N: Levels of Eotaxin (p = 0.001) and IL-4 (p = 0.08) were significantly higher at T3 compared to T1, and Eotaxin levels were also significantly higher at T3 as compared to T2 (p = 0.037). Levels of IFN-γ (p = 0.019), IL-1ra (p = 0.008) and MCP-1 (p = 0.008) were significantly higher, while PDGF-BB (p = 0.003) levels were significantly lower, at T2 as compared to T1.

Group I: Levels of G-CSF (p = 0.003), MIP-1β (p < 0.001), PDGF-BB (p < 0.001) and TNF-α (p = 0.024) were significantly decreased from T1 to T2. However, Eotaxin (p = 0.003) and IL-4 (p = 0.005) levels significantly increased from T2 to T3, and Eotaxin levels were also significantly increased at T3 when compared to T1 levels (p = 0.013). IFN-γ (p = 0.024) and MCP-1 (p = 0.001) levels were significantly increased at T3 compared to T1, while MIP-1α levels dropped significantly at T2 (p = 0.007) and T3 (p = 0.043). IL-6 levels were significantly lower at T3 compared to T1 (p = 0.005), and IL-9 levels were significantly lower at T2 compared to T1 (p = 0.043) and T3 (p = 0.043).

Group V: Eotaxin levels were significantly higher at T3 compared to T1 (p = 0.032). IFN-γ (p = 0.05 and < 0.001), IL-1ra (p = 0.001 and < 0.001) and MCP-1 (p = 0.032 and < 0.001) levels were significantly higher at T2 and T3 compared to T1. When compared to T1, IL-8 levels increased significantly at T2 (p = 0.032), while PDGF-BB levels demonstrated a significant drop during the same period (p < 0.001). IL-9 levels were significantly lower at T2 compared to T1 (p = 0.032) and T3 (p = 0.018) levels.

Group V+I: FGF-basic levels showed a significant drop between T1 and T3 (p = 0.026). Similarly, G-CSF (p = 0.014), IL-9 (p < 0.001) and MIP-1β (p = 0.007) levels also showed a significant drop from T1 to T2. MIP-1α (p = 0.007 and 0.029) and PDGF-BB (p < 0.001 and 0.029) levels dropped significantly at both T2 and T3 when compared to T1.
